# Designing Patient-Centered Text Messaging Interventions for Increasing Physical Activity Among Participants With Type 2 Diabetes: Qualitative Results From the Text to Move Intervention

**DOI:** 10.2196/mhealth.6666

**Published:** 2017-04-24

**Authors:** Gabrielle N Horner, Stephen Agboola, Kamal Jethwani, Aswita Tan-McGrory, Lenny Lopez

**Affiliations:** ^1^ Mongan Institute for Health Policy Massachusetts General Hospital Boston, MA United States; ^2^ Center for Connected Health Massachusetts General Hospital Boston, MA United States; ^3^ Disparities Solutions Center Massachusetts General Hospital Boston, MA United States; ^4^ Division of Hospital Medicine University of California San Francisco San Francisco, CA United States

**Keywords:** diabetes mellitus, type 2, text messaging, exercise, qualitative research

## Abstract

**Background:**

Type 2 diabetes mellitus (T2DM) is a disease affecting approximately 29.1 million people in the United States, and an additional 86 million adults have prediabetes. Diabetes self-management education, a complex health intervention composed of 7 behaviors, is effective at improving self-care behaviors and glycemic control. Studies have employed text messages for education, reminders, and motivational messaging that can serve as “cues to action,” aiming to improve glucose monitoring, self-care behaviors, appointment attendance, and medication adherence.

**Objectives:**

The Text to Move (TTM) study was a 6-month 2-parallel group randomized controlled trial of individuals with T2DM to increase physical activity, measured by a pedometer. The intervention arm received text messages twice daily for 6 months that were tailored to the participant’s stage of behavior change as defined by the transtheoretical model of behavior change.

**Methods:**

We assessed participants’ attitudes regarding their experience with text messaging, focusing on perceived barriers and facilitators, through two focus groups and telephone interviews. All interviews were audiorecorded, transcribed verbatim, coded, and analyzed using a grounded theory approach.

**Results:**

The response rate was 67% (31/46 participants). The average age was 51.4 years and 61% (19/31 participants) were male. The majority of individuals were English speakers and married, had completed at least 12th grade and approximately half of the participants were employed full-time. Overall, participants were satisfied with the TTM program and recalled the text messages as educational, informational, and motivational. Program involvement increased the sense of connection with their health care center. The wearing of pedometers and daily step count information served as motivational reminders and created a sense of accountability through the sentinel effect. However, there was frustration concerning the automation of the text message program, including the repetitiveness, predictability of text time delivery, and lack of customization and interactivity of text message content. Participants recommended personalization of texting frequency as well as more contact time with personnel for a stronger sense of support, including greater surveillance and feedback based on their own results and comparison to other participants.

**Conclusions:**

Participants in a theory-based text messaging intervention identified key facilitators and barriers to program efficacy that should be incorporated into future texting interventions to optimize participant satisfaction and outcomes.

**Trial Registration:**

Clinicaltrials.gov NCT01569243; http://clinicaltrials.gov/ct2/show/NCT01569243 (Archived by Webcite at http://www.webcitation.org/6pfH6yXag)

## Introduction

### Background

Type 2 diabetes mellitus (T2DM) is a disease affecting approximately 29.1 million people in the United States, and an additional 86 million adults have prediabetes [1]. Diabetes self-management education, a complex health intervention composed of 7 behaviors: (1) blood glucose self-monitoring, (2) taking medications, (3) healthy eating, (4) being active, (5) reducing risks, (6) healthy coping, and (7) problem solving, is effective at improving self-care behaviors and glycemic control [2]. Yet many individuals with T2DM fail to adopt and sustain a more active lifestyle despite its benefit on both health outcomes and quality of life [3-5].

Multiple meta-analyses have demonstrated overall success of mobile-based (mHealth) interventions with text messaging for glucose self-monitoring and glycemic control while results for long-term physical activity improvement among those with T2DM have been mixed [6-8]. However, mHealth is viewed as a promising intervention platform given the ubiquity of cell phones with texting and Internet access capabilities including among racial and ethnic groups and those with low socioeconomic status [9-11]. In fact, a 2015 Pew Research Center survey found that 92% of adults in the United States own a cell phone [12]. Importantly, mobile technologies can be tailored to an individual’s health behavior needs thus increasing the likelihood of initiating and maintaining lifestyle change [6]. Furthermore, text message-based studies have been shown to be a low-cost and feasible method of conveying health information because message banks can be predesigned and automatically sent to individuals on a set schedule. Studies have employed text messages for education, reminders, and motivational messaging that can serve as “cues to action,” aiming to improve glucose monitoring, self-care behaviors, appointment attendance, and medication adherence [6,13-17]. The Text to Move (TTM) program was a 2-parallel group randomized controlled trial that recruited participants with T2DM from health centers affiliated with a large academic center [18]. All individuals in the study were given an ActiPed+ pedometer—a small, wireless activity sensor—that recorded daily step count, distance traveled, calories burned, and activity time. Daily step counts were uploaded either manually or automatically (via Bluetooth wireless technology) onto a Web-based portal. The per subject, per month cost of TTM was approximately US $30.

Participants in the intervention arm were sent two messages a day for a 6-month period. An interdisciplinary team of physicians and health researchers created a bank of over 1000 text messages; subsequently, a behavioral psychologist grouped messages into the stages of behavior change specified by the transtheoretical model of behavior change [19,20]. The Transtheoretical Model states that behavior change is a nonlinear and dynamic process that involves 6 stages: precontemplation, contemplation, preparation, action, maintenance, and termination [21]. Participants’ stage of change was measured at enrollment and reevaluated every month, dictating the content of text messages sent. The messages were designed to provide bite-sized (160-character length) coaching based on daily step count, captured by pedometers, and preset physical activity goals that were agreed upon at the initial visit. The messages aimed to provide support, health education, motivation, and reminders to promote healthy behaviors. The text messages were designed at a 3rd grade reading level and were provided in English or Spanish, according to participant preference. Finally, to optimize engagement, two messages per week were question-based and required a simple response from participants. The TTM study design was first tested for participant acceptance in a 3-week feasibility study with 20 individuals [22].

### Purpose of the Study

Behavioral interventions should be theory-driven and take into account a participant’s beliefs, preferences, readiness for change, and perceived risks and benefits. Currently, there is a paucity of published studies that assess participants’ experiences with text messaging interventions, hindering the development of successful, patient-centered programs. The purpose of this study was to collect participants’ feedback from the TTM program in order to ascertain the facilitators and barriers to participant engagement. In addition, we inquired about (1) overall satisfaction and perceived effect of participating in the TTM program, (2) knowledge of and barriers to physical activity, (3) perception of text message frequency, content, style, and impact of texts on physical activity, (4) effect of wearing a pedometer, and (5) barriers to intervention efficacy.

## Methods

### Recruitment

The study was approved by the Institutional Review Board for the Massachusetts General Hospital and registered at clinicaltrials.gov/ct2/show/NCT01569243. A total of 98 individuals completed the TTM study of which 46 were assigned to the intervention arm and were contacted for poststudy interviews 6 months to 1 year after the study concluded.

### Study Procedures

All English-speaking TTM intervention participants were asked to participate in one of two focus groups held at two health care centers where participants were originally recruited. These focus groups spanned 90 min, were conducted in English and employed an in-depth, semi-structured guide (see Multimedia Appendix 1). Participants were compensated US $40 for attending.

If an individual did not attend a focus group or was Spanish-speaking, they were contacted for a telephone interview. Interviews were conducted either in English or Spanish by one of two trained researchers using an adapted questionnaire from the focus groups (see Multimedia Appendix 2) to facilitate telephone engagement. Interviews lasted at most 45 min and participants received US $25. The interview guide was professionally translated to Spanish and reviewed for accuracy by two native Spanish speakers. Saturation was reached when members of the study team determined participant responses were reoccurring and no new insights or information was being gathered [23,24].

### Data Analysis

All interviews and focus group sessions were audiorecorded, professionally transcribed verbatim, and then reviewed by team members for accuracy and completeness. Transcripts were analyzed with NVivo 9 (QSR International Pty Ltd, Version 9.2.81.0, 2010). A grounded theory approach was used in data analysis, beginning with two researchers reading three transcripts and identifying main themes [25]. Three additional transcripts were chosen based on richness of text, and four team members read through each transcript, modifying the codes, proposing subthemes, and drawing connections between codes. All four researchers conferred and discussed disagreements with our application of codes, ultimately finalizing a thematic framework through consensus. Using this framework, two researchers double-coded two additional transcripts and compared our results to ensure high intercoder reliability. In asking the same questions in the one-on-one interviews and focus groups, the content was comparable although it was not possible to quantify our results [25].

## Results

### Participation

**Figure 1:**
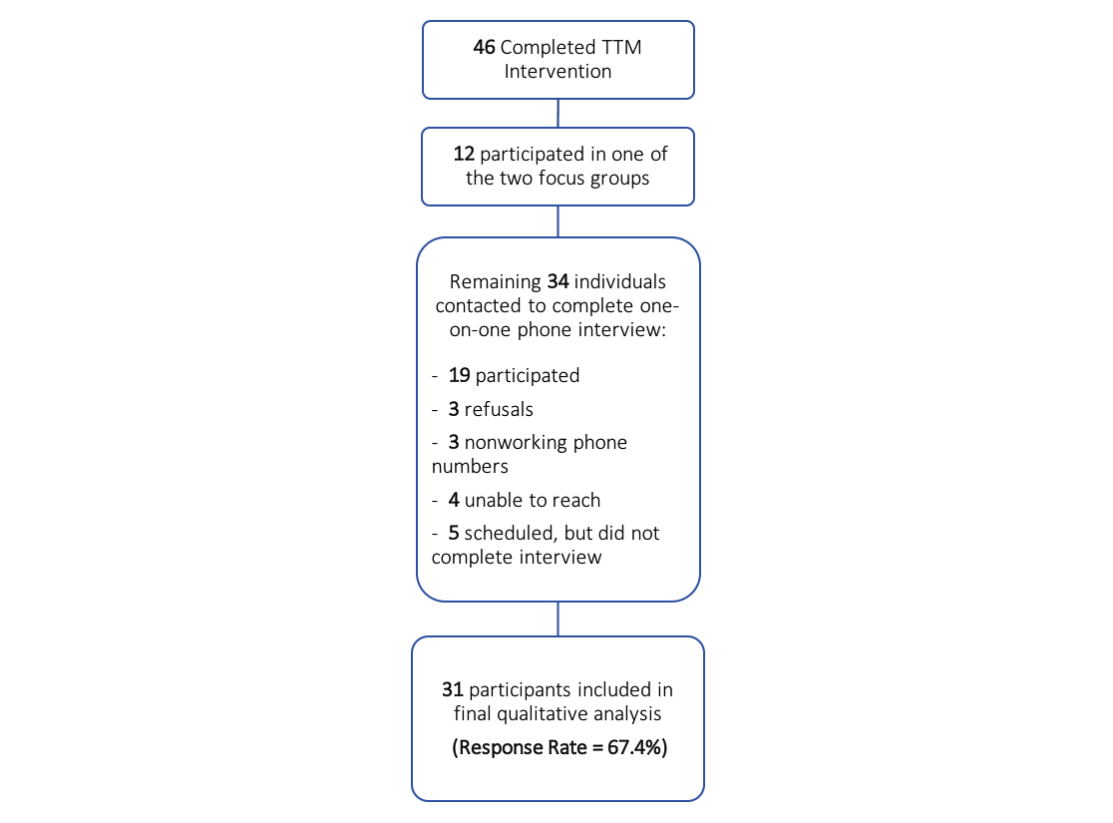
Recruitment of participants from Text to Move (TTM) intervention arm.

Figure 1 shows the recruitment of the 46 participants from the TTM intervention arm to attend a focus group or complete a phone interview 6 months to 1 year after the study concluded. For the focus groups, 38 English speakers were invited, 14 agreed to participate, and 12 individuals attended (7 in the first focus group and 5 in the second). Of the remaining 34 individuals who did not partake in a focus group, 24 agreed to participate in a phone interview and were scheduled, 19 individuals participated, and 4 were conducted in Spanish. In total, 31 of the 46 individuals who completed the TTM intervention participated in either a focus group (n=12) or a one-on-one interview (n=19), yielding an overall response rate of 67% (31/46 participants).

### Participant Characteristics

Of the 31 participants, 19 were male, and the average age was 51.4 years (see Multimedia Appendix 3 for full demographics table). The majority of individuals were married and English speakers, had completed at least 12th grade or attained a General Education Development (GED; ie, high school equivalency diploma). Approximately half of all participants were employed full-time. Compared with individuals who did not participate, participants in postintervention follow-up sessions were not significantly different in demographic characteristics.

### Thematic Analysis

On the basis of English and Spanish interviews, four domains emerged from our analysis: (1) effect of study participation, (2) effect of wearing the pedometer, (3) effect of text messages, and (4) barriers to intervention efficacy. The following are select quotes in conjunction with Textboxes 1-4.

**Domain 1: themes and subthemes with associated quotes.**

Effect of participating in Text to Move (TTM)

Increase in daily disease awareness

- *Diabetes is an everyday part of my life. Insulin, pills, of course exercise. I thought (TTM) was awesome, actually. It kind of reminded me every time I got a text.*

Program integrated into daily life

- *I mean, first describing it, it seems like, “Oh this may be a pain,” but once it’s just like everything else, once you implement it into your lifestyle it becomes second nature and it really wasn’t that bad.*

Connections arising from participation

- Increased connection and sense of support from health care team
  - *I did try to do the best that I could, so that the research could open up more doors or more windows to people to get the job done the right way, to help out others.*
  - *For me, (the program) did matter because it means that I was doing something that people were putting the time and money into it. Because even if it would have been that I didn’t get anything back, any positive feedback or a reminder, I would have been out. They don’t care. So if they don’t care, why should I care? But just by receiving those text messages on a daily basis and just seeing on the computer how good I was doing, it just kept giving more encouragement for me to do the right stuff.*
- Ambivalence or no change in connection nor sense of support from the health care team
  - *I wanted to believe that there was actually a person who gave a crap about what my data said. It didn’t feel that way.*

**Domain 2: themes and subthemes with associated quotes.**

Effect of wearing the pedometer and receiving texts on daily step count

Motivational through viewing step count and related texts

- *I’m very much like you where I need that instant—like I need it right now to show me. So with that (pedometer), when I would go home and see the numbers, that would motivate me, when I saw how close I was...I’d go outside at 11:40 (pm) because I knew I had 20 minutes.*
- *I was challenging myself to do better and to go farther than previous...I knew what I did the day before and was like, “How do I challenge myself to go a little bit farther, to push myself a little bit hard today?”*
- *It was very great to have it working correctly, and synchronized (to the) computer, and you could have the graph to see if you were up by a little, you know, how it worked, or your level, you know, how you were doing. A lot of people are visual. So when you see that visual in front of you, it’s very powerful.*

Sentinel effect from using the pedometer and receiving step count information

- *I’d feel guilty if I didn’t reach my goal everyday...I felt bad, like I’m not doing my homework.*
- *It made me feel motivated...I would (exercise) because I was afraid they were going to say, “Hey! Get off that sofa!”*

Evangelizing new technology to others not involved in study

- *I told all my friends actually, everybody was asking me, “What’s that in your shoes?” Because I put it in my shoes, and I told them that’s to make me move (chuckles).*

**Domain 3: themes and subthemes with associated quotes.**

Effect of text messages

Text messages as daily reminders

- *I never expected that it was a live person behind. I knew it was kind of like, this is six o’clock. I’m going to get the message, whether it’s the same one that I had before. I just knew it was like a tool and a reminder. I did find it like a reminder.*

Text messages as informational or useful for idea generation for increasing physical activity

- *There were some times that, let’s say I didn’t walk. They would be like, “Try to do this type of exercise”...They would give me an example of what other exercises I could have done or stuff like that. So it opens up your mind to see what other things you can do.*

**Domain 4: themes and subthemes with associated quotes.**

Barriers to Text to Move (TTM) intervention efficacy

- Technical issues
- Personal reasons
  - Lack of investment in the study
  - Comorbidities or physical pain prevented exercise
  - Environmental factors
    - *It would have been nice, like if it was raining out, we’re all in the same area, “Even though it’s raining out, you can still get active.”*
- Dislike of automation of text messages
  - Text messages were too repetitive and predictable
    - *I can tell that they were a can of limited messages that were repeating over and over again. It seemed like it was coming out and it was automated, almost like an alarm clock, that would give you a text. I would almost expect it.*
  - Inability to control text frequency
  - Lack of personalization
    - *The text messages, I thought, were too generic. I thought that they should be specifically more to your accomplishments, and not just the general daily thoughts of the people that are programming it.*
    - *(The messages) should specifically target that individual...relating to what you are achieving, or what you’re trying to achieve.*
  - Lack of interactivity of text messages
    - *You should be able to voice your opinion, which you can’t answer them back when they send you a message. That they should let you do.*
- Limited knowledge of recommended physical activity

#### Domain 1: Effect of Study Participation

##### Theme: Increase in Daily Diabetes Awareness

Overall, most respondents reported that participation increased awareness of the daily presence of diabetes in their lives and of their current levels of physical activity.

##### Theme: Program Integrated into Daily Life

Many participants explained that once they grew accustomed to wearing the pedometer and began to anticipate text messages, the program was incorporated into their daily routine.

##### Theme: Connections Arising From Participation

###### Increased Connection and Sense of Support From Health Care Team

Several participants exhibited high relationality within the TTM program, noting that their involvement made them feel as if “someone cared.” A focus group participant explained, “It felt like, yea, someone cares what I did today. Even though I have great support from my family, it’s my private, personal, little cheerleader.” Other respondents relayed feelings of “being a part of a team” during participation in the TTM study.

Furthermore, when asked if and how the program altered participants’ views of their clinician or health care center, many described developing a more positive outlook. In fact, approximately one-third of participants reported that it was their physician who introduced them to the program and encouraged them to enroll. One individual recollected, “If (doctors) mention it to (patients) in the right way, like my doctor did, you’d want to get involved. I believe my doctor’s the one who really made me feel comfortable about it.”

###### Ambivalence or No Change in Connection Nor Sense of Support From Health Care Team

Almost half of the participants demonstrated low relationality during the program. In other words, many felt that their involvement in the TTM program was arbitrary and they did not believe that anyone was monitoring their progress. When asked who they perceived to be sending the text messages, a quarter of respondents had no opinion of the sender. They expressed ambivalence when asked how the program impacted their view of their health care center or physician, stating that their health care provider was not aware of their involvement.

#### Domain 2: Effect of Wearing the Pedometer and Receiving Texts on Daily Step Count

##### Theme: Motivation Through Viewing Pedometer Step Count and Related Texts

For over half of participants, the pedometer proved to be a motivating tool, as step count was perceived as a tangible and realistic target. One female described, “...I would see my numbers and I’m like, ‘I’m so close to this goal.’ I would go out in my backyard and just walk in circles just to get to that next level.”

Specifically, “seeing the numbers” represented a goal that participants could attain. Many participants described that knowing the previous day’s step count provided a measure that could be reached or surpassed the following day. The Web-based portal was generally viewed favorably, as several participants noted that it was helpful to track progress longitudinally and observe patterns of physical activity.

##### Theme: Sentinel Effect From Using the Pedometer and Receiving Step Count Information

Over half of participants explained that they felt their performance in the TTM program was being monitored closely and thus they felt motivated to improve their levels of physical activity. This awareness of being monitored and the subsequent adjustment of behavior is known as the “sentinel effect.” For instance, one individual stated, “The majority of the time I was physically active because I knew I was being monitored, and I was trying to challenge myself.”

##### Theme: Evangelizing New Technology to Others Not Involved in Study

A few individuals were very enthusiastic about sharing the technology with family, friends, and colleagues.

#### Domain 3: Effect of Receiving the Text Messages

##### Theme: Text Messages as Daily Reminders

Most participants regarded the text messages as daily reminders about physical activity or a diabetes self-care behavior. This perception of the text messages as a functional tool, rather than a source of motivational or educational information, was very common. For example, a participant described, “It reminded me about taking my medication, it reminded me, ‘Yeah, I need to get up and go exercise.’ I always thought it would be good for quitting smoking, too.”

##### Theme: Text Messages as Informational and Useful for Idea Generation for Increasing Physical Activity

A few individuals recalled that the texts provided helpful hints on ways to increase physical activity. One participant recounted, “Instead of parking right next to the door at my work, I parked at the very end of the parking lot and walked. It was one of these things, those little reminders, ‘don’t park so close to your work,’ ‘don’t park so close to the mall entrance,’ ‘park farther away,’ and get in a few more minutes of walking a day.”

#### Domain 4: Barriers to Text to Move (TTM) Intervention Efficacy

##### Theme: Technical Issues

The most common complaint with the TTM program was malfunctioning of the pedometer or computer software, which led to illogical text messages being sent to participants. Technological issues were a source of frustration with a quarter of respondents. Two participants reported having trouble using their phone keypads to write a text, preventing a response to question-based messages.

##### Theme: Personal Reasons

###### Lack of Investment in the Study

Individuals often cited reluctance toward physical activity or diabetes self-management as reasons why TTM was not effective. For instance, a couple of participants revealed that they were not emotionally invested in the program. In discussing the text messages, one man said, “They weren’t supportive—they probably would have been supportive if I was willing to do stuff.”

###### Comorbidities or Physical Pain Prevented Exercise

About half of respondents explained that comorbidities or pain prevented physical activity. A few participants wished the text messages had taken into account the physical challenge associated with the transition period between a sedentary and active lifestyle. For example, “I kind of ignored (the text messages) because if I was hurting I would do nothing. I would just not pay attention to it.”

###### Environmental Factors

Many individuals reported that there were external factors inhibiting physical activity such as inclement weather, time constraints, or exhaustion from daily work requirements.

##### Theme: Dislike of Automation of Text Messages

###### Text Messages Were Too Repetitive and Predictable

Many participants divulged that the text messaging component became predictable, monotonous, and toward the end of the program they would ignore the texts. One male respondent elaborated, “When they’re repetitive, they were nagging because it’s like, ‘I already read this, I need something new’... it’s kind of sad, but at the end of it, it was like really annoying.”

###### Inability to Control Text Frequency

A few participants reported that they became weary of the unchanging frequency and timing of the text messages. Although over half of respondents “felt fine” with the number of the texts messages, a few said that there were too many. Several participants expressed interest in controlling the amount and timing of the messages that they received.

Important to note, whereas a few participants were dissuaded by the automation of the program, others did not mind the consistency of the text messaging system. One man stated, “To me it didn’t matter, because when you get a text message it’s so impersonal that it doesn’t matter who it comes from, where it comes from. As long as it says the right thing.” In discussing the amount and regularity of the text messages, one man explained, “I would say no, not annoyed. I like the word ‘nagging’ better. It was like motherly love.”

###### Lack of Personalization of Text Messages

There was frustration with the lack of personalization of the text messages. Several participants stated that they would be more likely to read the texts if they contained information specific to their health goals or progress. In addition to addressing activity goals, many participants agreed that they would like the text messages to include a personal touch, such as their name.

###### Lack of Interactivity of Text Messages

The lack of interactivity associated with the text messaging system was another source of dissatisfaction, participants often asserting, “there was no way you can respond.”

##### Theme: Limited Knowledge of Recommended Physical Activity

There was a lack of differentiation between physical activity and exercise. When probed during the one-on-one interview, only 4 participants identified the two as separate constructs. When questioned about the recommended level of physical activity for individuals with T2DM, approximately half of individuals said that they were not aware of the guidelines. A few respondents said that they received information on appropriate activity levels from their physician, whereas others said that they knew that physical activity was important, although they were not sure how much was necessary. When asked the most frequent form of physical activity that they partake in, half of participants replied, “walking,” whereas one-third of respondents answered, “use of equipment or weights.”

## Discussion

### Principal Findings

This study reports the perspectives of participants who completed a texting-based physical activity program for individuals with T2DM. TTM was a unique program for three significant reasons: (1) it combined pedometer and text-message components, (2) the text messages were developed based on the transtheoretical model of behavior change, and (3) participants could move between stages of change according to pedometer activity input. There was general participant satisfaction with the program, with over 90% of respondents agreeing that they would recommend the TTM program to a friend with some participants evangelizing the pedometer and TTM to others not involved in study. Yet, several themes reveal sources of frustration among study participants, including a lack of control over program intensity, impersonalization of text messages, and limited feedback from study coordinators. At the same time, we found that participants’ views on TTM were impacted both by social influences and personal reasons.

To start, participants varied in their preferences for program intensity stating that they wanted more control over texting frequency. Our finding is consistent with the variable-ratio schedule operant conditioning phenomenon in which a schedule of behavioral learning reinforcement is accomplished through an unpredictable sequence of communication or rewards. This schedule creates a steady and high rate of behavior change compared with a fixed schedule that was used in TTM [26]. Other studies have allowed participants to control the frequency and timing of text messages [6,27]. One texting-based weight loss program was designed to automatically reduce the number of text messages sent to a participant if their response rate was declining, aiming to minimize annoyance [28]. Texting-based interventions should utilize the variable-ratio schedule or permit greater participant autonomy in regulating text frequency in order to reduce texting fatigue and increase the likelihood of engagement.

In addition to the frequency pattern of messages, meta-analyses of technology-based studies suggest that personalization of message content can lead to higher participant retention and engagement [6,7]. Our results corroborate these findings. Tailoring encompasses three domains: personalization, feedback, and content matching (identifying determinants of a given behavior that an individual must focus on to achieve that behavioral outcome [29]). In content matching, potential barriers to physical activity could be identified at enrollment and text content adjusted to incorporate tailored solutions [30,31]. In addition, teaching problem solving techniques has been shown to be an essential component of behavior interventions and could be easily integrated into mHealth programs [29] enhancing participants’ self-efficacy and empowerment for long-term behavioral change [32]. TTM respondents recommended that messages could incorporate not only name and gender, but also the participant’s behavioral preferences and goals, perceived barriers, previous text responses, and medical status. Through personalizing text messages, participants could feel “coached” as opposed to “hassled,” potentially increasing their attention to messages. Importantly, the feasibility of personalization has been demonstrated in several texting-based studies that rely on low-cost computer programming to formulate messages [32,33].

Immediate feedback is also considered a cornerstone of a tailored invention as it is used to validate and encourage participants’ progress [34-36]. TTM was characterized by mostly unidirectional, computer-generated communication that many participants found exasperating. However, other participants reported increased connections arising from participation and were very pleased with the program’s design. Evidence of high relationality included perceived programmatic or clinical support, being “a part of a team” and the “sentinel effect” (ie, the tendency for performance to improve when participants become aware that their behavior is being evaluated and believe that meaningful consequences could follow). Our results suggest that through amplifying the “human component” via interactivity, the monotony of automated messaging, often necessary due to feasibility and cost restrictions, could perhaps be overcome. For example, CareSmarts, a successful diabetes behavioral modification intervention, combined computer-generated text messaging with remote nursing support [27]. The interactive nature of the text messages allowed for immediate nurse follow-up if a health problem arose. Although we were unable to correlate relationality with outcomes, previous studies link participant awareness of “someone on the other end” to greater participant activation, retention, higher self-efficacy (ie, an individual’s belief that a behavior-specific goal can be achieved), and greater physical improvements, including a larger reduction in HbA1c (glycated hemoglobin) [7,27,35,37-39]. It is important to consider that requiring a response from participants may entail a higher level of mobile phone proficiency. At the same time, expanding the role of program staff may increase social support and subsequently improve diabetes self-management [40]. There is limited evidence that text messages are a sufficient stand-alone tool to engage patients in long-term, complex behavioral change and our findings highlight the value of maintaining human interaction in a technology-based program [41].

Identified themes pertaining to preference for increased relationality align with the unified theory of acceptance and use of technology (UTAUT), which outlines several factors influencing technology adoption: effort expectancy (perceived ease of use of the technology); performance expectancy (perceived usefulness of the technology); and social influences (perceived expectations from others regarding one’s personal use of the technology [42]). Effort expectancy is essential to technology adoption and future studies will need to offer varying degrees of continual technical support. Problems with technology was the most frequent complaint among participants and perhaps contributed to disengagement with the program. Our analysis also revealed that performance expectancy and social influence shaped participants’ perceptions of the TTM program, particularly if they felt connected to their health care provider and clinic. Prior studies have found that participation over time in mHealth interventions can be improved through regular reminders from clinicians and that there is a patient preference for occasional in-person interaction with physicians [43,44]. mHealth interventions should underscore perceived support from clinical team members and further investigate optimal contact intensity for program success. This is critical when considering the importance of end-user satisfaction in increasing long-term success [45].

Finally, it is important to recognize that T2DM is a multifaceted condition affected by a range of behavioral and physiological processes. The theme describing text messages as reminders, which while useful to many, reveals that TTM may have been too narrow in scope to promote long-term behavioral change. Unlike interventions aimed at achieving smoking cessation or hand washing, programs that seek to increase physical activity levels may require a more comprehensive approach [46,47]. Several texting-based studies that address physical activity along with supplemental behaviors such as diet, tobacco use, and diabetes self-care activities, have demonstrated greater health outcomes and lower attrition rates [48,49]. Other studies involving individuals with T2DM have successfully integrated a glucometer with a mobile device to monitor blood glucose levels [45,50]. There are several behaviors implicated in T2DM outcomes and interventions should reflect this complexity. Prior non-mHealth physical activity behavioral interventions have included additional features that may be of use in mHealth-based studies: (1) goal setting; (2) behavioral contracting; and (3) adjustable physical activity goals that are personalized to a participant’s stage of change [49].

### Limitations

Despite study strengths, our results should be interpreted with the following limitations. First, interviews were conducted half a year to one year following closeout of the 6-month TTM program. Therefore, recall and social desirability biases may have impacted participant responses to be more favorable than if collected during or immediately after the intervention. However, we are reassured that the data collected during focus groups and one-on-one interviews, though spaced months apart, revealed common themes. Finally, our results may not be generalizable to other settings because our participants were recruited from community health centers aligned within a large, urban academic health center in Massachusetts. Participants were mostly English-speaking and had high levels of educational attainment and employment.

### Comparison With Prior Work

Texting-based interventions have the potential to improve diabetes self-management and enhance physical activity in individuals with type 2 diabetes. Future studies should prioritize participant involvement and input in the design of texting features [51]. In addition, enhancing the human connection, via in-person or telephone contact, while also providing personal feedback and problem solving guidance may increase program efficacy, as participants are made to feel accountable and supported during the study. Finally, including supplemental health behaviors in the content of text messages, such as diet and medication adherence, may aid in participant engagement.

### Conclusions

Although text messaging represents a scalable and cost-effective method of facilitating communication between patients and their health care team [52], our results suggest that texting should not substitute, but rather supplement clinical support. In all, twice-daily text messages paired with a pedometer device were generally accepted as congruous tools to motivate and monitor physical activity for individuals with type 2 diabetes.

## Appendix

Multimedia Appendix 1 Guide for focus group discussions.

Multimedia Appendix 2 Guide for one-on-one exit interviews.

Multimedia Appendix 3 Characteristics of participants versus nonparticipants in post-intervention interviews.

## Abbreviations

GED: General Education Development high school equivalency diploma
.gov: Government Web domain
HbA1c: glycated hemoglobin
mHealth: mobile-based health interventions
T2DM: type 2 diabetes mellitus
TTM: Text to Move
UTAUT: unified theory of acceptance and use of technology
www: World Wide Web
