# Supplementary material for: Designing Patient-Centered Text Messaging Interventions for Increasing Physical Activity Among Participants With Type 2 Diabetes: Qualitative Results From the Text to Move Intervention
Source: JMIR Mhealth Uhealth. 2017 Apr 24;5(4):e54. doi: 10.2196/mhealth.6666 (PMC5422654; doi:10.2196/mhealth.6666)
Supplement: Multimedia Appendix 1 [file mhealth_v5i4e54_app1.pdf]

Multimedia Appendix I: Guide for focus group discussions

Discussion Outline:

1. Tell us your name, what city you live in, and one effect diabetes has had on your daily life.
2. What was the main reason you decided to participate in the Text to Move program?
3. What is the first thought that comes to your mind when you think of physical activity?
4. How would you define physical activity?
    a. Is exercise different than physical activity?
    b. What types of physical activity or exercise are a part of your everyday life?
    c. Were some activities more difficult than others?
5. What do you know about the recommended amount of physical activity for diabetes patients?
6. What kinds of physical discomforts, if any, did you experience while being physically active?
    a. Did any concern you or prevent you from exercising?
7. On days that you were physically active, what motivated you?
8. On days that you were not physically active, what prevented you from exercising?
9. Think back to when you were receiving text messages daily. How did you feel about the text messages you received?
10. How important was the content of the messages? Did what they say matter, or did they primarily function as reminders?
11. What types of messages did you find to be the most helpful? Unhelpful? Surprising? Confusing? [Slide show]

12. In the context of your daily lives, when did the text messages typically come?
    a. When would be the best time to receive text messages?
13. Overall, did you feel the text-messages were more supportive or more nagging? Why?
14. How do you feel about the number of text messages you received?
    a. Did you find yourself ignoring or getting annoyed by text messages after a while? If so, at what point?
15. Who did you imagine was sending you the text messages?
    a. What was their intention?
    b. Did you feel that anyone was looking at the messages?
    c. How would you characterize the person(s) sending and/or reading the messages?
    d. Did this change over time?
    e. Did you ever feel like you might let someone down or be judged if you failed to meet your physical activity goals?
16. What affect, if any, did the text messages have on your view of your clinic?
    a. Alt: What affect, if any, did participation in the Text to Move program have on your view of your clinic?
17. What affect, if any, did the text messages have on your relationship with your medical provider (i.e., your nurse, nurse practitioner, or doctor)?
    a. Alt: What affect, if any, did your participation in the Text2Move program have on your relationship with your medical provider?
18. Are you still exercising as much as you were during the program? Why or why not?

19. If a friend who was thinking about participating in the Text to Move program asked you what the pros and cons were, what would you say?

a. Alt: How would you describe the program to a friend thinking about participating in the Text to Move program?

Closing (10 minutes)

- Closing remarks
- Summary and “Did we miss anything?” question
- Thank the participants
- Issue their compensation if available.

Well, that’s all the questions I have for you tonight. Thank you very much for your feedback. It was very helpful.
