# Supplementary material for: Designing Patient-Centered Text Messaging Interventions for Increasing Physical Activity Among Participants With Type 2 Diabetes: Qualitative Results From the Text to Move Intervention
Source: JMIR Mhealth Uhealth. 2017 Apr 24;5(4):e54. doi: 10.2196/mhealth.6666 (PMC5422654; doi:10.2196/mhealth.6666)
Supplement: Multimedia Appendix 2 [file mhealth_v5i4e54_app2.pdf]

Multimedia Appendix II: Guide for one-on-one exit interviews

Participant Interview Protocol

The purpose of this study is to identify ways you think the Text to Move Program may have motivated you to exercise regularly.

Your answers will be confidential – your name and any identifying information will be kept separate from your responses. I'd like to audio record the interview so that it can be transcribed later. The recording will only be used for research purposes and will only be analyzed by study researchers.

As an incentive for participating, we'll send you a $25 Visa gift card. At the end of the interview, I'll ask for your address so that I can mail it to you.

Confirm informed consent sent and permission to record the interview.

I'd like to begin the interview by getting some background information about your health status and why you decided to participate in Text to Move.

1. What effect has diabetes had on your daily life?
2. What was the main reason you decided to participate in the Text to Move program?

These next questions are about exercise.

3. What is the first thought that comes to your mind when you think of physical activity?
4. How would you define physical activity?
    a. Is exercise different than physical activity?
    b. What types of physical activity or exercise are a part of your everyday life?
    c. Were some activities more difficult than others?
5. What do you know about the recommended amount of physical activity for diabetes patients?
6. What kinds of physical discomforts, if any, did you experience while being physically active?
    a. Did any concern prevent you from exercising?
7. On days that you were physically active, what motivated you?

8. On days that you were not physically active, what prevented you from exercising?

Now I'd like you to think back to when you were receiving Text to Move messages.

9. When you were receiving text messages daily, how did you feel about the text messages you received?

10. How important was the content of the messages? Did what they say matter, or did they primarily function as reminders?

11. What types of messages did you find to be the most helpful? Unhelpful? Surprising? Confusing?

12. In the context of your daily life, when did the text messages typically come?

   a. When would be the best time to receive text messages?

13. Overall, did you feel the text-messages were more supportive or more nagging? Why?

14. How do you feel about the number of text messages you received?

   a. Did you find yourself ignoring or getting annoyed by text messages after a while? If so, at what point?

15. something like, "Who do you think would be best to send the text messages?" Followed by "Did you have an idea who might have sent the messages?" "What would be different about the text messages if (whoever they said) sent you the message?"

16. Did your Primary Care Physician talk with you at all about Text to Move? Was he or she aware that you were participating in the program?

17. What affect, if any, did the text messages have on your view of your clinic?

   a. Alt: What affect, if any, did participation in the Text to Move program have on your view of your clinic?

18. What affect, if any, did the text messages have on your relationship with your medical provider?

   a. Alt: What affect, if any, did your participation in the Text to Move program have on your relationship with your medical provider?

19. Are you still exercising as much as you were during the program? Why or why not?

20. If a friend who was thinking about participating in the Text to Move program asked you what the pros and cons were, what would you say?
    a. Alt: How would you describe the program to a friend thinking about participating in the Text to Move program?

This last question will complete the interview.

21. Is there anything about your experience with Text to Move that you'd like to add?

Thank you for taking the time to talk with me about your experiences and the Text to Move. We appreciate your participation.

What address would you like us to use to mail you your $25 Visa Gift Card? (Confirm Address)
