# Supplementary material for: Designing Patient-Centered Text Messaging Interventions for Increasing Physical Activity Among Participants With Type 2 Diabetes: Qualitative Results From the Text to Move Intervention
Source: JMIR Mhealth Uhealth. 2017 Apr 24;5(4):e54. doi: 10.2196/mhealth.6666 (PMC5422654; doi:10.2196/mhealth.6666)
Supplement: Multimedia Appendix 3 [file mhealth_v5i4e54_app3.pdf]

Characteristics of participants versus nonparticipants in post-intervention interviews			
Characteristics	Participated in focus group or interview<br>% (n) (n=31)	Did not participate in focus group or interview<br>% (n) (n=15)	*P* value
**Sex**			.93
Female	39% (12)	40% (6)	
Male	61% (19)	60% (9)	
**Ethnicity**			.20
White	74% (23)	53% (8)	
Hispanic	19% (6)	20% (3)	
Black	3% (1)	13% (2)	
Asian or Pacific Islander	3% (1)	13% (2)	
**Language**			.66
English	87% (27)	80% (12)	
Spanish	13% (4)	20% (3)	
**Employment**			.40
Unemployed	16% (5)	7% (1)	
Employed full-time	52% (16)	53% (8)	
Employed part-time	13% (4)	20% (3)	
Student	0% (0)	7% (1)	
Homemaker	7% (2)	0% (0)	
Disabled	10% (3)	0% (0)	
Retired	3% (1)	7% (1)	
Other	0% (0)	7% (1)	
**Education**			.55
1st to 8th grade	3% (1)	7% (1)	
9th to 11th grade	7% (2)	7% (1)	
12th grade or GED[a]	52% (16)	33% (5)	
1-3 years of college	26% (8)	47% (7)	
≥4 years of college	13% (4)	7% (1)	
**Marital status**			.19
Married	61% (19)	47% (7)	
Living with partner	13% (4)	7% (1)	
Divorced or separated	23% (7)	20% (3)	
Single (never married)	3% (1)	13% (2)	
Widowed	0% (0)	13% (2)	

[a]General Education Development (GED) is a high school equivalency diploma.
